# Hypervariable intronic region in *NCX1 *is enriched in short insertion-deletion polymorphisms and showed association with cardiovascular traits

**DOI:** 10.1186/1471-2350-11-15

**Published:** 2010-01-28

**Authors:** Katrin Kepp, Elin Org, Siim Sõber, Piret Kelgo, Margus Viigimaa, Gudrun Veldre, Neeme Tõnisson, Peeter Juhanson, Margus Putku, Andreas Kindmark, Viktor Kožich, Maris Laan

**Affiliations:** 1 Institute of Molecular and Cell Biology, University of Tartu, Tartu, Estonia; 2 Centre of Cardiology, North Estonia Medical Centre, Tallinn, Estonia; 3 Tallinn University of Technology , Department of Biomedical Engineering, Chair of Medical Physics, Tallinn, Estonia; 4 Department of Cardiology, University of Tartu, Tartu, Estonia; 5 Department of Medical Sciences, Uppsala University Hospital, Uppsala, Sweden; 6 Institute of Inherited Metabolic Diseases, Charles University - First Faculty of Medicine, Prague, Czech Republic

## Abstract

**Background:**

Conserved non-coding regions (CNR) have been shown to harbor gene expression regulatory elements. Genetic variations in these regions may potentially contribute to complex disease susceptibility.

**Methods:**

We targeted CNRs of cardiovascular disease (CVD) candidate gene, *Na(+)-Ca(2+) exchanger (NCX1) *with polymorphism screening among CVD patients (n = 46) using DHPLC technology. The flanking region (348 bp) of the 14 bp indel in intron 2 was further genotyped by DGGE assay in two Eastern-European CVD samples: essential hypertension (HYPEST; 470 cases, 652 controls) and coronary artery disease, CAD (CADCZ; 257 cases, controls 413). Genotype-phenotype associations were tested by regression analysis implemented in PLINK. Alignments of primate sequences were performed by ClustalW2.

**Results:**

Nine of the identified *NCX1 *variants were either singletons or targeted by commercial platforms. The 14 bp intronic indel (rs11274804) was represented with substantial frequency in HYPEST (6.82%) and CADCZ (14.58%). Genotyping in Eastern-Europeans (n = 1792) revealed hypervariable nature of this locus, represented by seven alternative alleles. The alignments of human-chimpanzee-macaque sequences showed that the major human variant (allele frequency 90.45%) was actually a human-specific deletion compared to other primates. In humans, this deletion was surrounded by other short (5-43 bp) deletion variants and a duplication (40 bp) polymorphism possessing overlapping breakpoints. This indicates a potential indel hotspot, triggered by the initial deletion in human lineage. An association was detected between the carrier status of 14 bp indel ancestral allele and CAD (*P *= 0.0016, OR = 2.02; Bonferroni significance level alpha = 0.0045), but not with hypertension. The risk for the CAD development was even higher among the patients additionally diagnosed with metabolic syndrome (*P *= 0.0014, OR = 2.34). Consistent with the effect on metabolic processes, suggestive evidence for the association with heart rate, serum triglyceride and LDL levels was detected (*P *= 0.04).

**Conclusions:**

Compared to SNPs targeted by large number of locus-specific and genome-wide assays, considerably less attention has been paid to short indel variants in the human genome. The data of genome dynamics, mutation rate and population genetics of short indels, as well as their impact on gene expressional profile and human disease susceptibility is limited. The characterization of *NCX1 *intronic hypervariable non-coding region enriched in human-specific indel variants contributes to this gap of knowledge.

## Background

Cardiovascular disease (CVD) is a complex disorder affecting heart and blood vessels, which develops from the interaction between life style patterns and genetic susceptibility to the disease. Western societies face high and increasing rates of CVD (such as coronary artery disease, hypertension, arteriosclerosis, heart failure and arrhytmia etc.), which is considered a number one cause of premature death and disability. Although CVD has been shown to have significant heritability, pinpointing of the genes and variants associated with the elevated risk to the disease has been challenging [1,2]. The focus has slowly switched from DNA variants located in genic regions causing direct changes in the encoded protein to the regulatory variants affecting gene expression. Non-coding variants potentially contributing to the susceptibility to complex diseases are localized in promoters and enhancers, introns or *5'*- and *3'-UTR*s, and may affect binding of the gene expression regulators, such as transcription and splicing factors or miRNAs. Comparative genetics studies have noted several essential gene regulatory elements that are conserved among species [3,4]. Thus, targeting evolutionarily conserved non-coding regions (CNR) in candidate genes for CVD may pinpoint regulatory elements directing the gene expression profile. Genetic variation in these regions may contribute to the susceptibility to CVD. Based on these hypotheses we aimed to target human CVD candidate gene *Na(+)-Ca(2+) exchanger (NCX1; SLC8A1) *with polymorphism screening in CNRs and to test associations of identified variants with CVD and related metabolic traits in two Eastern-European populations.

Na^+^/Ca^2+ ^exchange participates in the regulation of vascular function and thus, disturbances in this process contribute to the development of CVD. Na^+^/Ca^+2 ^exchanger (NCX1) is a bidirectional calcium transporter, responsible for calcium homeostasis in cardiac myocytes and in other cell types by catalyzing the exchange of one Ca^2+ ^ion for three Na^+ ^ions across plasma membrane [5]. Altered Na^+^/Ca^2+ ^exchange activity has been observed in arrhythmias, heart failure [6], and salt-sensitive essential hypertension [7,8]. *Ncx1*^-/- ^mice showed complete lack of Na^+^/Ca^2+ ^exchange activity in heart leading to the defects in heart development and embryonic lethality [9].

*NCX1 *gene (498 908 bp) is located in chromosome 2p22.1 and consists of 12 alternatively spliced exons[10]. Alternative splicing of *NCX1 *produces several tissue-specific isoforms [11] differing in their regulatory properties [12,13]. NCX1 alternative isoforms respond differently to potential therapeutic agents such as polyunsaturated fatty acids [14] and specific NCX1 inhibitors [9]. Currently, genetic studies targeting the association of *NCX1 *polymorphisms with CVD are limited. Resequencing of the entire coding and promoter regions in Japanese population identified 15 polymorphisms, two of these variants located >23 kb upstream of the mRNA transcription start site were associated with hypertension [8].

We have conducted a polymorphism screening in *NCX1 *non-coding regions. The most potential genetic variant to affect gene function, a 14 bp indel, localized in an intronic hypervariable region was characterized in detail in cardiovascular and metabolic traits in two European populations.

## Methods

### In silico analysis of conserved non-coding regions (CNR) of NCX1

We screened *NCX1 *(also known as *SLC8A1*) locus for the presence of Conserved Non-coding Regions (CNRs) using the web-based VISTA software [15] with the proposed default parameters (cutoff criteria: 100 bp sliding window; sequence identity 70%; comparison with rat and mouse). The analyzed *NCX1 *locus (in total 420,181 bp) spanned from 10 kb downstream to 10 kb upstream of the gene [2p22.1; coordinates 40 241 046-40 661 226 according to NCBI Build 35, hg17; GenBank:6546]. All VISTA regions that had any overlap with annotated genes track at UCSC Genome Browser [16] were excluded as potential coding regions. For polymorphism discovery we selected 29 non-coding regions based on the following criteria: the (i) length 50-300 bp; (ii) location >200 bp from the nearest exons, and (iii) sequence identity >70% between human and both rodents (See additional file 1).

### DHPLC screening of novel polymorphisms in NCX1 non-coding regions

The selected *NCX1 *conserved non-coding segments were targeted to polymorphism screening by *Denaturing High-Performance Liquid Chromatography *method (DHPLC; Wave Technologies Inc. USA). During the design and experimental screening process of DHPLC products the recommendations of the manufacturer were followed. Details of DHPLC assay and running conditions with appropriate PCR primers and fragment characteristics are given in additional file 2. Among the total 29 CNR-s selected, 16 intronic regions entered the DHPLC screening (See additional file 1) phase. 13 regions were excluded before the laboratory experiments due to failure in DHPLC primer design, inappropriate length of the PCR and CNR fragment (too long >700 bp or short <50 bp), or more than two different Tm melting points for the region of interest. The average length of the screened CNR segments was 163 bp (SD: 64 bp, range: 70-287 bp) and PCR fragments was 334 bp (SD: 87 bp, range 170-489 bp). Polymorphism screening was performed with 15 different DNA pools, each consisting of DNAs of three patients with cardiovascular disease originating from two Eastern European sample sets (n = 22 from HYPEST and n = 24 from CADCZ study; detailed description is given below). Individual DNAs in the pools exhibiting evidence for the presence of a polymorphism were sequenced at least twice on both forward and reverse orientations.

### DGGE genotyping assay

Genotyping of the identified 14 bp indel (rs11274804, NCBI dbSNP database) in *NCX1 *intron 2 was performed by standard Denaturing Gradient Gel Electrophoresis (DGGE) (Ingeny, Goes, Netherlands). The manufacturer's recommendations were followed in the design of the DGGE assay and in choosing the conditions for the experimental setup. Detailed information of the assay is given in additional file 2. To initially validate the reliability of the DGGE assay, all DNA samples with alternative genotypes previously detected by DHPLC were re-genotyped at the DGGE platform. To further assure DGGE gel typing system's quality in each assay, double positive (product containing 14 bp indel) and negative controls were used. All ambiguous genotypes in DGGE analysis were re-genotyped twice and/or sequenced on both DNA strands by an ABI 377 Prism automated DNA sequencer using ReproGel 377 gels (Amersham Biosciences Inc., USA). The sequences of all novel variants were verified by resequencing twice on both forward and reverse orientation.

### Subjects for association studies with cardiovascular disease

Two Eastern-European sample collection, HYPEST and CADCZ were used to conduct association analysis of rs11274804 with cardiovascular traits and serum biomarkers (Table 1). The HYPEST study has been approved by the Ethics Committee on Human Research of University of Tartu (no. 122/13, 22.12.2003; 137/20, 25.04.2005). CADCZ study was approved by the Ethics Committee of Charles University--1st Faculty of Medicine (December 1996) and is published elsewhere [17]. The studies were carried out in compliance with the Helsinki Declaration and all the participants have given their written informed consent. These sample collections have been recruited to target the genetic-epidemiological component of cardiovascular disease in Estonian and Czech populations, respectively. HYPEST subjects were recruited across Estonia during 2004-2007 (1823 individuals, age range 18-85 years) with the aim to evaluate risk factors for essential hypertension and related cardiovascular disease. Details of the recruitment are given in additional file 2. CADCZ subjects (n = 893; n = 296 coronary artery disease patients, n = 597 controls) were recruited by the Cardiology Department of the 2^nd ^Clinic of Internal Medicine, Faculty Hospital Královské Vinohrady in Prague Czech Republic and Czech heath clinics in years 1998-2000 [17]. In order to exclude obesity and age-related risks, the current study included individuals with BMI <35 kg/m^2 ^and age <65 years. From HYPEST individuals 470 hypertensive patients and 652 normotensive controls, and from CADCZ samples 257 CAD patients and 413 controls, were analyzed. The control group for both studies consisted of matched healthy individuals with no personal history of CAD, essential hypertension, MI, peripheral arterial disease, or stroke. As no population differentiation was detected among HYPEST and CADCZ study subjects previously [18], the controls of the two studies were pooled in order to address the association of rs11274804 with cardiovascular traits in general Eastern-European population.

**Table 1:** Phenotypic characteristics of analyzed individuals

	HYPEST		CADCZ		Healthy Eastern
Variable	Cases	Controls	Cases	Controls	European individuals^1^
No. of individuals	470	652	257	413	1065
*Parameters *(mean ± SD):					
Age (years)^2^	43.9 (13.0)	39.0 (4.9)	51.2 (8.1)	49.5 (7.4)	43.7 (9.7)
Body Mass Index (BMI) (kg/m^2^)	28.7 (3.7)	24.4 (3.3)	27.9 (3.3)	25.3 (3.1)	24.7 (3.1)
Systolic blood pressure (SBP) (mmHg)	143.2 (17.6)	130.0 (22.7)	136.6 (19.2)	125.2 (14.0)	128.5 (12.2)
Diastolic blood pressure (DBP) (mmHg)	87.4 (10.6)	81.0 (14.6)	85.1 (11.3)	80.3 (9.3)	80.0 (8.2)
Total cholesterol (mmol/liter)	5.6 (1.1)	5.0 (1.4)	5.4 (1.0)	5.6 (1.0)	5.4 (1.1)
High-density lipoprotein (HDL) (mmol/liter)	1.5 (0.4)	1.7 (0.5)	1.2 (0.3)	1.5 (0.4)	1.5 (0.4)
Low-density lipoprotein (LDL) (mmol/liter)	3.8 (1.0)	3.3 (1.1)	3.2 (0.8)	3.4 (0.9)	3.3 (0.9)
Triacylglycerols (mmol/liter)	1.8 (1.6)	0.8 (0.3)	2.1 (1.3)	1.5 (1.1)	1.2 (1.1)
Intima media thickness (IMT)	NA	NA	0.7 (0.2)	0.6 (0.2)	NA
Heart Rate (HR) (bpm)	NA	71.0 (26.4)	76.0 (5.6)	74.8 (5.5)	74.0 (8.6)
Medication:					
% of antihypertensive treatment	75.3%	0%	54.5%	0%	0%
% of antilipidemic treatment	20.2%	0%	58.4%	0%	0%

^1^Pooled HYPEST and CADCZ control subjects, who had no personal history of cardiovascular disease, including essential hypertension, myocardial infarction, coronary artery disease, stroke, and had never been prescribed antihypertensive or other cardiovascular medications. Previously, no population differentiation was detected between HYPEST and CADCZ study subjects[18]

^2^Cases: age at the onset of the essential hypertension (HYPEST) or coronary artery disease (CADCZ); Controls: age at the recruitment

mmHg - millimeters of mercury; bpm - beats per minute; NA - not available

### Cardiovascular phenotype

For all subjects in the HYPEST and CADCZ studies resting blood pressure (BP) and heart rate were measured by trained clinicians during recruitment. In both studies BP measurements per subject were obtained after a rest in a sitting position using a standard mercury column sphygmomanometer and size-adjusted cuffs. All HYPEST individuals possessed a documented history of multiple SBP and DBP readings (on average 4.31 readings per individual, range 2-29) during mean 3.17 years (range 1-17 years). To compensate for the variability in heaviness of data per study subjects, we used for the analysis the median across the longitudinal BP readings as well as the median of the subject's age during the readings. Definition of essential hypertension among HYPEST subjects is given in additional file 2. For CADCZ subjects three blood pressure measurements were documented and the median value was recorded.

Coronary artery disease (CAD) in CADCZ study was diagnosed according to WHO criteria, and one or more large stenosis of a major coronary vessel was confirmed by coronarography in all patients details of which have been published elsewhere [17]. Carotid wall intima media thickness (IMT) and the presence of carotid plaque, recorded in the CADCZ subjects were determined by ultrasonography using linear exploring coil 7,5 MHz on the distant interior wall about 1-2 cm distally from the bifurcation. The measurement was performed on the right and the left carotid 5-10 times on each side. Diagnosis of metabolic syndrome was defined based on the criteria appointed by International Diabetes Federation [19].

### Laboratory measurements

Altered serum lipid profile is considered as a cardiovascular risk factor - a condition that is associated with an increased risk of developing CVD affecting the heart and blood vasculature. In the current study, lipid measurements (total-cholesterol, HDL-cholestrerol, LDL-cholesterol and triglycerides, TG) were determined from fasting venous blood samples in the HYPEST and CADCZ subjects. For HYPEST total-cholesterol, HDL-cholesterol, LDL-cholesterol, and triglycerides in the serum were measured by standardized assays (Cobas Integra 800^® ^analytical platform, Roche Diagnostics, Inc., USA) at the United Laboratories, Tartu University Hospital [20] or at the Diagnostics Division Laboratory, the North Estonia Medical Centre [21]. For CADCZ, serum lipids were measured by standard techniques in Institute of Clinical Chemistry of Vinohrady Faculty Hospital.

### Statistical analysis

For all identified polymorphisms, the deviation from Hardy-Weinberg equilibrium and differences in allele frequencies between populations were tested using an exact test implemented in Genepop web Version 3.4 [22]. Four rare population-specific polymorphisms showed differences in allele frequencies between the studied individuals from HYPEST (n = 22) and CADCZ (n = 24) (Fisher's exact test, p < 0.05; data not shown). There was no significant difference between the HYPEST (n = 1122) and CADCZ (n = 670) study groups (Fisher's exact test, p > 0.05; data not shown) for the distribution of the 14 bp indel polymorphism.

The significance of the associations between the *NCX1 *14 bp indel and cardiovascular traits was tested, and odds ratios/effect sizes and confidence intervals were obtained using linear (quantitative traits) and logistic (case-control analysis) regression analyses implemented in the PLINK software [23]. In all study stages the association analyses were performed under additive and dominant genetic models. Additive genetic models assume a trend per copy of the minor allele to contribute to the trait or disease susceptibility on genotype categories, whereas dominant genetic models assume that heterozygotes have the same increased risk as minor homozygous genotypes. Recessive genetic model was not applied, as it requires a large sample size to reach a reasonable statistical power. Meta-analysis was performed using inverse variance method with fixed effect model of both sample sets. Association tests were performed with age, sex and BMI as co-variates. *P*-values <0.05 were considered statistically significant. Two additional corrections were used in the quantitative parameter analysis: in the serum lipid biomarker analysis, a correction according to Jun Wu was implemented to all of the individuals obtaining lipid-lowering medications [24], and in the blood pressure (SBP and DBP) association test, a correction described by Martin Tobin was used for all subjects receiving antihypertensive treatment [25]. For the multiple comparisons (11 traits tested) a simple Bonferroni correction was used (p < 0.05/11 = 0.0045).

Multiple sequence alignment of the human, common chimpanzee and rhesus macaque DNA sequences of the orthologous regions adjacent to 14 bp indel within *NCX1 *intron 2 was performed with web-based analysis tool ClustalW2 [26]. LD structure (based on the HapMap variation data) for the analyzed *NCX1 *genomic region was performed with the Haploview package [27] (See in additional file 3).

## Results

### DHPLC screening of polymorphisms in NCX1 non-coding regions

We analyzed human cardiovascular candidate gene, *NCX1 *(*Na(+)-Ca(2+) exchanger*, 2p22.1), for the conserved non-coding regions using VISTA genome browser [15] with default parameters. In total, we identified 365 conserved non-coding regions between human and mouse and rat (May 2004, NCBI Build 33). These conserved non-coding regions (CNR) covered 15.1% of the analyzed genomic region (gene ± 10 kb). Based on the length (50-300 bp), location (>200 bp from the nearest exon) and sequence conservation (>70% between human and both rodents), 29 regions were selected for further polymorphism screening among 46 East-European cardiovascular disease patients (HYPEST n = 22; CADCZ n = 24). Due to the limitations of the technology and/or complex structure of the DNA sequence, 13 of the selected *NCX1 *regions did not qualify for the DHPLC screening. The remaining 16 regions were subjected to polymorphism discovery among cardiovascular phenotype patients. Detailed descriptions of the selected regions as well as inclusion/exclusion criteria for the analysis are given in additional file 1. In total, ten genetic variants (including three novel variants) were identified within the 16 analyzed regions (Table 2). Most of the variations were located within the second intron of the gene (first intron relative to ATG initiation codon), including six SNPs, and one 14 bp indel (rs11274804, NCBI dbSNP database). Two SNPs were detected in the *NCX1 *intron 10 and one SNP in intron 4. Among the screened HYPEST samples (n = 22) three common (minor allele frequency, MAF>10%) and four rare (MAF<10%) variants were identified. Five polymorphisms detected in the studied CADCZ patients (n = 24) were uncommon (MAF<10%) and four were common (MAF>10%). Four variants were specific to one of the studied sample sets (either HYPEST or CADCZ).

**Table 2:** Polymorphisms detected by DHPLC in screened human *NCX1 *non-coding regions

Analyzed regions		Detected variants^1^					
Contig positions in Chr. 2^2^	Length (bp)	Location	Alleles^3^	HYPEST (hom/het)^4^	CADCZ (hom/het)^4^	Validation/ rs-number^2^	Targeted by genotyping platform^5^
40349616	293	intron 2	A/g	ND	1/4	novel	NA
40335702-40335701	348	intron 2	14 bp indel^6^	0/3	0/7	rs11274804^6^	NA
40335650	348	intron 2	C/g	0/1	0/1	novel	NA
40407194	387	intron 2	C/g	0/1	1/3	rs72943138	Illumina
40277948	370	intron 2	T/c	3/6	0/2	rs449383	Affymetrix GeneChip
40475254	469	intron 2	A/t	3/9	0/1	rs2192773	Illumina
40301091	292	intron 2	a/G	ND	1/1	rs2540904	Illumina
40246617	282	intron 4	T/c	ND	0/1	novel	NA
40514809	489	intron 10	A/g	3/9	0/5	rs4952414	Illumina
40514961	489	intron 10	c/T	0/1	ND	rs17026003	Affymetrix GeneChip

^1^Polymorphism screening was performed using HYPEST (n = 22) and CADCZ (n = 24) cases. Detailed description of all targeted genomic regions and detected variants is given in additional file 1.

^2^Contig positions and rs-numbers of the validated SNPs are given according to the Simple Nucleotide Polymorphisms database (dbSNP build 129; Human Genome March 2006).

^3^Major and minor alleles are indicated with capital and lower case letters, respectively.

^4^Number of number of homozygotes/heterozygotes (hom/het) of each identified polymorphism among the screened individuals

^5^Commercial genotyping platforms, which include the described variants (according to NCBI database)

^6^the 14 bp indel: CATTCCCTCTCCAT/-

ND - not detected; NA - not available

### Characterization of the intronic hypervariable region

Nine of the identified variants in screened *NCX1 *non-coding regions were either singletons or targeted already by commercial platforms and thus included in large number of studies (Table 2). The current study focused on the 14 bp indel (rs11274804), which was represented with substantial frequency (6.82% in HYPEST; 14.58% in CADCZ) in both study populations. In addition, the location of rs11274804 indel variant in the second intron of *NCX1 *(the first intron in the coding region) raised the hypothesis about its potential effect on the gene transcription as several gene expression regulatory elements have been mapped within the first introns [28-30]. Thus, this polymorphism as well as its flanking region was selected for further characterization.

**Figure 1:**
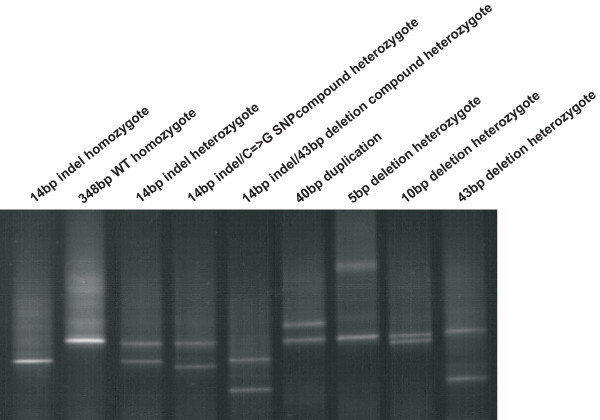
**Detection of alternative genotype variants of human *NCX1 *intron 2 studied region (348 bp) by Denaturing Gradient Gel Eelectorphoresis (DGGE)**. DGGE was performed using 9% polyacrylamide gel in 0.5 × TAE buffer containing 30-85% denaturing gradient of ureumformamide. Electrophoresis conditions were 58°C, 12 h and 140 V.

**Figure 2:**
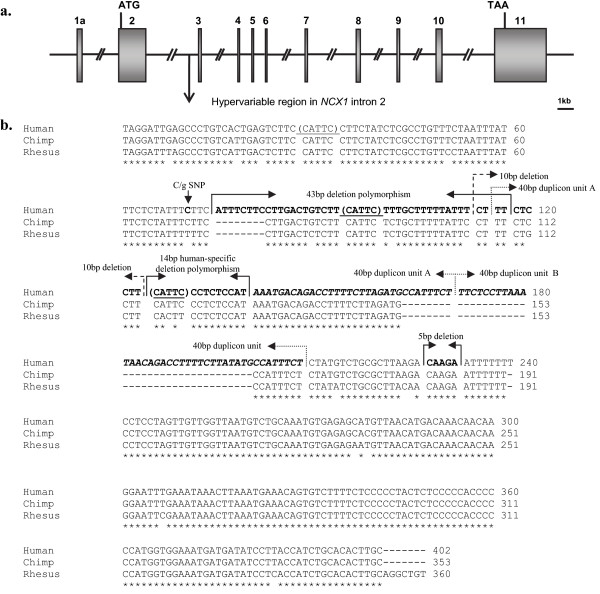
**The human *Na(+)-Ca(2+) exchanger (NCX1) *gene**: **(a) **Genomic structure shown in arbitrary scale (adapted from [8]; (**b**) Hypervariable region within intron 2 (348 bp): identified human variants mapped on human-chimpanzee-macaque DNA sequence alignment. The human polymorphisms (C = >G SNP, 43 bp deletion, 10 bp deletion, 40 bp duplication, 14 bp indel and 5 bp deletion) are highlighted by arrows and **in bold**. Duplicons A and B of the 40 bp duplication are indicated *in italics*. Indel hotspot motifs GTAAG (reverse strand: CATTC; [36,43] are underlined and enclosed in the brackets.

The region flanking the 14 bp indel (348 bp) in *NCX1 *intron 2 was targeted for the larger-scale genotyping using Denaturing Gradient Gel Electrophoresis (DGGE). Our study samples, HYPEST (n = 1122) and CADCZ (n = 670), displayed nine different genotype variants of this intronic segment (representing seven novel alternative alleles), detected by DGGE and confirmed by sequencing (Figure 1). The analyzed region appeared to be highly polymorphic in both sample collections. In addition to 14 bp indel, a SNP (C/G), a duplication of 40 bp segment, and four alternative deletions (10 bp, 43 bp and 5 bp) were localized within the 348 bp region (Figure 1, Table 3). The breakpoints of several detected variants co-localized with the 14 bp indel (Figure 2b). The allele frequencies of the detected variants in the full genotyped sample (n = 1792) varied from singletons (10 bp deletion; 40 bp duplication) to common polymorphisms with allele frequencies up to 8.51% (14 bp indel). The 43 bp deletion was enriched in HYPEST samples (20 subjects in HYPEST versus 1 in CADCZ). One HYPEST subject appeared to be a compound heterozygote for 14 bp indel/43 bp deletion. In total, 18.21% of the genotyped HYPEST subjects and 20.49% of CADCZ subjects were carrying alternative variants of the studied *NCX1 *intron 2 segment (Table 3). Active genome dynamics of the analyzed region is supported by its location between two LD-blocks within the *NCX1 *gene (See in additional file 3).

**Table 3:** Distribution of insertion/deletion variants identified in the *NCX1 *intron 2 hypervariable region

	HYPEST		CADCZ		
	Cases	Controls	Cases	Controls	All
Number of studied individuals	470	652	257	413	1792
*Detected genotypes*^1 ^(n, %):					
WT homozygote	379 (80.6%)	513 (78.7%)	192 (74.7%)	356 (86.2%)	1440
14 bp indel heterozygote	68 (14.5%)	117 (18.9%)	55 (21.4%)	53 (12.8%)	293
14 bp indel/C = >G compound heterozygote	8 (1.7%)	8 (1.2%)	5 (1.9%)	3 (0.7%)	24
14 bp indel homozygote	3 (0.6%)	1	2 (0.8%)	1	7
43 bp del heterozygote	10 (2.1%)	10 (1.5%)	1	0	21
40 bp duplication	1	0	0	0	1
14 bp indel/43 bp del compound heterozygote	0	1	0	0	1
5 bp del heterozygote	1	2 (0.3%)	1	0	4
10 bp del heterozygote	0	0	1	0	1

^1^Exact sequences of detected alleles are given in Figure 2.

n = number of carriers of the genotype; indel - insertion/deletion; del - deletion

### Identified 14 bp indel originates from a human-specific deletion compared to ancestral primate sequence

In order to determine the ancestral primate variant of the studied 348 bp region, the consensus sequences of human, common chimpanzee and rhesus macaque were aligned. In contrast to the expectations, multiple sequence alignment (ClustalW2) revealed that the ancestral primate variant is actually the minor human allele carrying the 14 bp sequence motif. The major human variant (among East-European subjects) has evolved through a 14 bp human-specific deletion when compared to sister-species chimpanzee and rhesus macaque (Figure 2b).

Other identified short insertion-deletion variants within the studied region have occurred in human lineage on the chromosomal variant carrying the human-specific 14 bp deletion (Figure 2b).

### Association of the 14 bp indel with cardiovascular traits

Association of *NCX1 *intronic 14 bp indel (rs11274804) with cardiovascular disease was studied in two Eastern European sample sets: essential hypertension (HYPEST, Estonia; n = 1122; cases n = 470/controls = 652) and coronary artery disease, CAD (CADCZ, Czech; n = 670; cases n = 257/controls = 413). Associations were assessed using logistic regression under additive and dominant effect models (age, sex, and BMI as covariates; Table 4). *NCX1 *intronic 14 bp indel region revealed strong association with the diagnosis of CAD (*P *= 0.0016, OR = 2.02; *P *= 0.0018, OR = 2.07; additive and dominant models, respectively). As there are various clinical conditions that comprise CAD, additional case-control analysis was performed using patients diagnosed with CAD as well as metabolic syndrome (n = 88). Despite a three fold reduction in sample size compared to the full CADCZ patient group, the analysis of CAD patients with metabolic syndrome revealed highly significant association with increased effect size (*P *= 0.0014, OR = 2.34; *P *= 0.0016, OR = 2.41; additive and dominant models, respectively). These associations remained significant after correction for multiple testing (Bonferroni significance level α = 0.05/11 = 0.0045).

**Table 4:** Association between cardiovascular disease and *NCX1 *intronic 14 bp indel rs11274804

			Minor allele		Association testing using logistic regression^1^			
			frequency (%)		Additive model		Dominant model	
			
Disease	Sample	Sample size cases/ controls^2^	Cases	Controls	P-value	OR [95%CI]	P-value	OR [95%CI]
Essential hypertension	HYPEST	470/652	7.76	9.04	0.14	0.70 [0.44, 1.12]	0.09	0.65 [0.40, 1.06]
Coronary artery disease	CADCZ	257/413	12.45	7.02	**0.0016**	2.02 [1.30, 3.13]	**0.0018**	2.07 [1.31, 3.26]
Coronary artery disease & metabolic syndrome	CADCZ	88/361	15.07	7.64	**0.0014**	2.34 [1.38, 3.96]	**0.0016**	2.41 [1.39, 4.18]

^1^Logistic regression analysis was performed with the following covariates: sex, age, BMI.

^2^Detailed definition of cases and controls for essential hypertension, coronary artery disease and metabolic syndrome is given in Materials and Methods, as well as in additional file 2.

Significant differences have been highlighted in bold, **P < 0.05**.

*NCX1 *intronic 14 bp indel was not associated with the diagnosis of essential hypertension in HYPEST sample collection (*P *> 0.1).

### Association of the 14 bp indel with quantitative cardiovascular parameters

Associations of the 14 bp indel with quantitative cardiovascular parameters [systolic (SBP) and diastolic (DBP) blood pressure, heart rate, Intima-Media Thickness (IMT)], and serum lipid biomarkers [total cholesterol, HDL, LDL, triglycerides (TG)] were evaluated by linear regression under additive and dominant models (Table 5, Table 6). A marginal negative correlation was detected with heart rate (*P *= 0.04, beta = -1.6; additive model) and LDL (*P *= 0.04, beta = -0.26; dominant model) among healthy Eastern-European subjects (Table 5, Table 6). Association of the 14 bp indel with serum triglyceride levels reached marginal significance in the CADCZ sample (*P *= 0.04, beta = 0.25; both models) and a non-significant trend for association in the joint meta-analysis with HYPEST data (*P *= 0.08, beta = 0.18; *P *= 0.07, beta = 0.19; additive and dominant models, respectively). No significant association was detected with other studied cardiovascular and serum lipid parameters in separate sample sets and in meta-analysis.

**Table 5:** Association between cardiovascular parameters and the carrier status of the 14 bp indel in *NCX1 *intron 2

						Association testing using linear regression^1^			
			WT/WT	WT/ indel	indel/ indel	Additive model		Dominant model	
			
	Sample	n	Mean (± SD)	Mean (± SD)	Mean (± SD)	P-value	Effect (SE)	P-value	Effect (SE)
SBP (mmHg)^2^	HYPEST	997	140.7 (21.19)	141.4 (22.93)	168.7 (36.14)	0.31	1.50 (1.47)	0.45	1.14 (1.52)
	CADCZ	670	132.4 (20.29)	134.1 (22.00)	135.0 (18.03)	0.44	1.43 (1.85)	0.42	1.54 (1.92)
	Meta-analysis	1667	137.4 (21.23)	138.4 (22.80)	151.8 (31.51)	0.20	1.47 (1.15)	0.28	1.29 (1.19)
	Healthy subjects^3^	1048	128.5 (5.66)	128.0 (10.53)	136.5 (9.19)	0.47	-0.71 (0.98)	0.39	-0.86 (0.99)
DBP (mmHg)^2^	HYPEST	993	86.9 (12.67)	87.2 (13.31)	104.3 (22.28)	0.39	0.82 (0.95)	0.57	0.55 (0.98)
	CADCZ	669	84.2 (11.51)	84.1 (15.45)	86.7 (11.55)	0.71	0.39 (1.07)	0.73	0.38 (1.11)
	Meta-analysis	1662	85.8 (12.29)	85.9 (14.29)	95.5 (18.59)	0.38	0.63 (0.71)	0.51	0.48 (0.74)
	Healthy subjects^3^	1048	81.4 (1.41)	80.5 (7.85)	85.0 (7.07)	0.11	-1.06 (0.66)	0.08	-1.15 (0.67)
Heart rate (bpm)	CADCZ	670	75.1 (5.51)	76.2 (5.83)	69.3 (3.06)	0.15	0.79 (0.54)	0.07	1.03 (0.56)
	Healthy subjects^3^	833	75.0 (8.49)	73.3 (8.06)	70.0 (0.00)	**0.04**	-1.61 (0.79)	**0.05**	-1.60 (0.80)
IMT (mm)	CADCZ	670	0.63 (0.20)	0.65 (0.20)	0.87 (0.29)	0.20	0.03 (0.02)	0.33	0.02 (0.02)
	CADCZ controls	413	0.58 (0.17)	0.54 (0.15)	1.20 (0.00)	0.42	-0.02 (0.02)	0.18	-0.03 (0.02)

^1^For association analysis with SBP and DBP regression testing for a linear trend of marker alleles was performed with age, sex and BMI as covariates. Association analysis with heart rate was performed with sex as covariate, and intima-media thickness without covariates.

^2^Correction for antihypertensive treatment was implemented to all treated patients as described[25].

^3^Pooled HYPEST and CADCZ control subjects, who had no personal history of cardiovascular disease, including essential hypertension, myocardial infarction, coronary artery disease, stroke, and had never been prescribed cardiovascular medications. Previously, no population differentiation was detected between HYPEST and CADCZ study subjects[18]

n - number of individuals; IMT - Intima-media thickness; Significant differences have been highlighted in bold, **p < 0.05**

**Table 6:** Association between serum lipid biomarkers and the carrier status of the 14 bp indel in *NCX1 *intron 2

						Association testing using linear regression^1^			
			WT/WT	WT/indel	indel/ indel	Additive model		Dominant model	
			
	**Sample**^2^	n	Mean (± SD)	Mean (± SD)	Mean (± SD)	P-value	Effect (SE)	P-value	Effect (SE)
Total cholesterol	HYPEST	459	5.92 (1.15)	5.85 (1.24)	6.40 (2.47)	0.86	-0.02 (0.14)	0.73	-0.05 (0.15)
(mmol/L)	CADCZ	670	5.50 (1.04)	5.48 (0.99)	5.55 (0.83)	0.11	0.17 (0.11)	0.14	0.17 (0.11)
	Meta-analysis	1129	5.67 (1.10)	5.63 (1.10)	5.98 (1.71)	0.25	0.10 (0.09)	0.33	0.09 (0.09)
	Healthy subjects^3^	431	5.59 (1.07)	5.36 (0.98)	6.37 (0.00)	0.18	-0.19 (0.14)	0.14	-0.22 (0.15)
HDL (mmol/L)	HYPEST	458	1.52 (0.42)	1.46 (0.42)	1.38 (0.29)	0.23	-0.06 (0.05)	0.24	-0.06 (0.05)
	CADCZ	670	1.41 (0.39)	1.41 (0.42)	1.42 (0.29)	0.81	-0.009 (0.04)	0.82	-0.01 (0.04)
	Meta-analysis	1128	1.45 (0.41)	1.43 (0.42)	1.40 (0.26)	0.35	-0.03 (0.03)	0.37	-0.03 (0.03)
	Healthy subjects^3^	431	1.52 (0.40)	1.61 (0.45)	1.70 (0.00)	0.10	0.09 (0.05)	0.10	0.09 (0.06)
LDL (mmol/L)	HYPEST	459	4.04 (1.03)	4.04 (1.00)	4.66 (2.58)	0.73	0.04 (0.12)	0.88	0.02 (0.13)
	CADCZ	651	3.33 (0.89)	3.22 (0.85)	3.22 (0.63)	0.66	-0.09 (0.20)	0.57	-0.12 (0.21)
	Meta-analysis	1110	3.63 (1.01)	3.54 (1.00)	3.94 (1.85)	0.94	0.01 (0.11)	0.87	-0.02 (0.11)
	Healthy subjects^3^	427	3.41 (0.91)	3.13 (0.85)	3.94 (0.00)	0.06	-0.23 (0.12)	**0.04**	-0.26 (0.13)
Tri-glycerides	HYPEST	458	1.77 (1.66)	1.82 (1.02)	1.29 (0.53)	0.99	0.003 (0.19)	0.89	0.03 (0.20)
(mmol/L)	CADCZ	670	1.72 (1.19)	1.94 (1.27)	2.00 (1.16)	**0.04**	0.25 (0.12)	**0.04**	0.26 (0.12)
	Meta-analysis	1128	1.74 (1.40)	1.89 (1.18)	1.65 (0.90)	0.08	0.18 (0.10)	0.07	0.19 (0.10)
	Healthy subjects^3^	431	1.52 (1.12)	1.40 (0.81)	1.62 (0.00)	0.44	-0.11 (0.15)	0.42	-0.12 (0.15)

^1^For association analysis with serum lipids regression testing for a linear trend of marker alleles was performed with age, sex and BMI as covariates.

^2^Correction for the treatment with lipid-lowering medication was implemented as described[24].

^3^Pooled HYPEST and CADCZ control subjects with available records for serum lipids. The individuals had no personal history of cardiovascular disease, including essential hypertension, myocardial infarction, coronary artery disease, stroke, and had never been prescribed cardiovascular medications. Previously, no population differentiation was detected between HYPEST and CADCZ study subjects[18]

n - number of individuals; Significant differences have been highlighted in bold, **p < 0.05**

## Discussion

We subjected the human *NCX1 *(*Na*^+^/*Ca*^2+^*exchanger*) gene to polymorphism screening in conserved non-coding regions with the aim to identify novel potential regulatory variants, which may contribute to the development of cardiovascular disease (CVD). So far, fine-scale polymorphism discovery in the coding, and promoter regions of the *NCX1 *gene have been carried out only among Japanese individuals, where twopromoter SNPs were shown to be associated with essential hypertension [8] as one of the major risk factor for several CVDs (i.e. CAD). We conducted the polymorphism discovery in *NCX1 *non-coding conserved regions using CVD patients from two Eastern-European sample collections (HYPEST, essential hypertension; CADCZ, coronary artery disease). Among the ten identified variants, the genomic context of the 14 bp indel located in *NCX1 *gene intron 2 and its association with CVD was studied in detail. First introns have previously been indicated to contain essential regulatory elements and therefore, may contribute to the transcriptional regulation processes [31] and splicing. For example, in human *CFTR *gene a regulatory intronic DNase I hypertensive site (DHS) was shown to be required for the normal expression levels in the intestinal epithelium *in vivo *[28]. The expression profiles of human angiotensin II type 2 receptor *AGTR2 *and erythroid-specific *GATA-1 *are affected by regulatory elements in intron 1 containing transcription factor binding sites [29,30].

Genotyping of the 14 bp indel locus revealed the hypervariable nature of the studied genomic segment within *NCX1 *intron 2. Among the screened Eastern-Europeans (n = 1792) the analyzed 348 bp region was represented by seven different alleles (Figure 1, Figure 2). The alignments of human-chimpanzee-macaque sequences revealed that the major human variant (allele frequency 90.45%) was actually a human-specific deletion compared to other primates. The most common alternative variant, the 14 bp indel, appeared to have the ancestral status among primates. Both, chimpanzee and rhesus macaque possess this common 14 bp sequence tract in their *NCX1 *intron 2 (Figure 2b). Either natural selection or genetic drift may have contributed to the enrichment of the novel 14 bp deletion variant among humans. The data suggests that the novel deletion variant may carry a selective advantage among humans as it was found to be associated with decreased risk for CAD and elevated serum triglyceride levels. In primate evolution, emergence of such short indel variants and indel-related transcriptional and translational changes may have provided an additional source for the flexible response of genomes to the changing life-style and environmental conditions. As a supportive observation, an enrichment of indels in immunity-associated loci has been found as a possible response to variable virus infections (i.e. HIV) in human and chimpanzee [32].

The human-specific deletion variant is surrounded by an abundance of other short (5-43 bp) deletion variants and a duplication (40 bp) polymorphism, which possess overlapping breakpoints (Figure 2b). This observed high variation refers to a potential indel hotspot, which may have been triggered by the initial 14 bp deletion in human lineage. This scenario is consistent with a recent report revealing the mutagenic role of the indel heterozygosity to its surrounding sequences [33]. The state of indel heterozygosity is expected to affect localized chromosome pairing during meiosis. Regions with indel heterozygosity might be prone to double stranded DNA breaks and are thus targeted to mutational repair, which in turn leads to higher mutation rate [33,34]. A consensus sequence motif GTAAG has been reported with the high prevalence within genomic regions prone to insertion/deletion events [35,36]. The sequence (CATTCCCTCTCCAT) of the 14 bp indel identified in this study contains the inverted sequence of this previously described hotspot motif on the reverse strand (GTAAG vs. CATTC). In addition, the studied hypervariable *NCX1 *intronic region harboured two further CATTC motifs (Figure 2b). Low LD in this region additionally refers to active genome dynamics (See in additional file 3).

High prevalence of small indels (<100 bp) has been found to be preferably associated with alternatively spliced genes, where partial inclusion and deletion of genic regions may broaden gene expression profiles in different tissue types [32,36]. Among the 12 exons coded by the human *NCX1 *gene there are six alternative untranslated 5'-exons denoted 1a-1f. Different combinations of these six exons may result in up to 32 different Na^+^/Ca^2+ ^exchanger mRNA transcripts [37]. The hypothesis that preferences in splice-site selection among *NCX1 *exons 1a-1f (and other alternative exons) may be affected by the genetic composition in the hypervariable intron 2 requires experimental proof.

The knowledge about the involvement of small indels (<100 bp) in increasing/decreasing susceptibility to the development of complex disease is still scarce. A well-known example is 32 bp deletion in human the *CCR5 *gene that results in a frameshift and premature termination [38,39]. This variant, which is common among Europeans (MAF 9.2%) and almost absent among Africans contributes to the resistance against HIV-1 infection. It was speculated that a 10 bp direct repeat that flanks the deleted region promoted a recombination event leading to the 32 bp deletion [39]. The effect of indels in non-coding regions is less understood. A recent study identified a 25 bp deletion in intron 32 of the human *MYBPC3 *gene leading to the loss of exon 33. The carriers of this deletion develop heritable cardiomyopathies and have increased risk of heart failure in Indian populations [40].

The current study identified a strong association between the carrier status of *NCX1 *intronic 14 bp indel and increased risk to coronary artery disease (CAD) in the East European population. The risk was the highest among the CAD patients with metabolic disease. Consistent with the effect on metabolic processes, the 14 bp indel was also associated with serum triglyceride levels. Several recent studies have highlighted the role of metabolic factors and metabolic syndrome in determining the extent of CAD and the risk for new vascular events [41,42]. In perspective, the association study of rs11274804 as well as other identified indel variants in *NCX1 *intron 2 with CAD and related metabolic factors is to be extended to other populations. The expression of *NCX1 *alternative transcripts in vascular muscle (NCX1.3 isoform) and in heart (NCX1.1 isoform) further supports the potential involvement of *NCX1 *genetic variants in susceptibility to coronary artery disease [7]. Functional studies would reveal whether the carrier status of alternative *NCX1 *intronic indel variants affects the alternative transcript profile of the gene.

## Conclusions

In summary, compared to SNPs targeted by large number of locus-specific and genome-wide assays, considerably less attention has been paid to short insertion-deletions (indels) variants in the human genome. The data of genome dynamics, mutation rate and population genetics of short indels, as well as their impact on gene expressional profile and human disease susceptibility is limited. The characterization of *NCX1 *intronic hypervariable region enriched in human-specific indel variants contributes to this gap of knowledge.

## Competing interests

The authors declare that they have no competing interests.

## Authors' contributions

KK contributed to the study design, performed polymorphism screening, genotyping and resequencing experiments, contributed to the analysis and interpretation of the data and write the first draft of the manuscript. EO was responsible for the collection of the HYPEST study sample, contributed to the analysis and interpretation of the data, and manuscript writing. SS contributed to the analysis, and manuscript writing. PK was responsible for preparing genomic DNAs of HYPEST and CADCZ study subjects, and assisted in DGGE genotyping. MV and GV contributed to the recruitment of HYPEST essential hypertension patients along with relevant clinical and epidemiological data. NT conducted the VISTA analysis. PJ and MP assisted in collection of HYPEST individuals. AK contributed to the DHPLC polymorphism screening. VK coordinated the recruitment of CADCZ study subjects. ML directed the study design and performance, the recruitment of HYPEST study samples and finalized the manuscript. All authors read and approved the final manuscript.

## Pre-publication history

The pre-publication history for this paper can be accessed here:

http://www.biomedcentral.com/1471-2350/11/15/prepub

## Supplementary Material

Additional file 1 **Characteristics of analyzed DHPLC and DGGE regions**. All analyzed *NCX1 *gene regions for DHPLC and DGGE assays. Characteristics of 29 regions: their location, conservation, PCR primers and product length, found polymorphisms and the reason for exclusion form the study. Click here for file

Additional file 2 **Additional information for Materials and Methods**. Additional information for Materials and Methods. Click here for file

Additional file 3 **LD structure of the human *NCX1 *gene 348 bp region**. LD structure of the human *NCX1 *gene region (chr2; position: 40 241 046-40 661 226) shown as r^2^-blot. Upper white bar marks the positions of HapMap SNPs. Both arrows indicate the location of 14 bp indel (rs11274804) between two LD-blocks in the second intron of the *NCX1 *gene. Click here for file

## References

[B1] Mohlke KL Boehnke M Abecasis GR Metabolic and cardiovascular traits: an abundance of recently identified common genetic variants Hum Mol Genet 2008 17 R2 R102 108 10.1093/hmg/ddn275 18852197 PMC2570060

[B2] Delles C McBride MW Padmanabhan S Dominiczak AF The genetics of cardiovascular disease Trends Endocrinol Metab 2008 19 9 309 316 10.1016/j.tem.2008.07.010 18819818

[B3] Dermitzakis ET Reymond A Antonarakis SE Conserved non-genic sequences - an unexpected feature of mammalian genomes Nat Rev Genet 2005 6 2 151 157 10.1038/nrg1527 15716910

[B4] Drake JA Bird C Nemesh J Thomas DJ Newton-Cheh C Reymond A Excoffier L Attar H Antonarakis SE Dermitzakis ET Conserved noncoding sequences are selectively constrained and not mutation cold spots Nat Genet 2006 38 2 223 227 10.1038/ng1710 16380714

[B5] Blaustein MP Physiological effects of endogenous ouabain: control of intracellular Ca2+ stores and cell responsiveness Am J Physiol 1993 264 6 Pt 1 C1367 1387 8392793 10.1152/ajpcell.1993.264.6.C1367

[B6] Schillinger W Fiolet JW Schlotthauer K Hasenfuss G Relevance of Na+-Ca2+ exchange in heart failure Cardiovasc Res 2003 57 4 921 933 10.1016/S0008-6363(02)00826-X 12650870

[B7] Iwamoto T Kita S Zhang J Blaustein MP Arai Y Yoshida S Wakimoto K Komuro I Katsuragi T Salt-sensitive hypertension is triggered by Ca2+ entry via Na+/Ca2+ exchanger type-1 in vascular smooth muscle Nat Med 2004 10 11 1193 1199 10.1038/nm1118 15475962

[B8] Kokubo Y Inamoto N Tomoike H Kamide K Takiuchi S Kawano Y Tanaka C Katanosaka Y Wakabayashi S Shigekawa M Association of genetic polymorphisms of sodium-calcium exchanger 1 gene, NCX1, with hypertension in a Japanese general population Hypertens Res 2004 27 10 697 702 10.1291/hypres.27.697 15785003

[B9] Iwamoto T Kita S Uehara A Imanaga I Matsuda T Baba A Katsuragi T Molecular determinants of Na+/Ca2+ exchange (NCX1) inhibition by SEA0400 J Biol Chem 2004 279 9 7544 7553 10.1074/jbc.M310491200 14660663

[B10] Kraev A Chumakov I Carafoli E The organization of the human gene NCX1 encoding the sodium-calcium exchanger Genomics 1996 37 1 105 112 10.1006/geno.1996.0526 8921376

[B11] Quednau BD Nicoll DA Philipson KD Tissue specificity and alternative splicing of the Na+/Ca2+ exchanger isoforms NCX1, NCX2, and NCX3 in rat Am J Physiol 1997 272 4 Pt 1 C1250 1261 9142850 10.1152/ajpcell.1997.272.4.C1250

[B12] Dunn J Elias CL Le HD Omelchenko A Hryshko LV Lytton J The molecular determinants of ionic regulatory differences between brain and kidney Na+/Ca2+ exchanger (NCX1) isoforms J Biol Chem 2002 277 37 33957 33962 10.1074/jbc.M206677200 12118014

[B13] Hurtado C Prociuk M Maddaford TG Dibrov E Mesaeli N Hryshko LV Pierce GN Cells expressing unique Na+/Ca2+ exchange (NCX1) splice variants exhibit different susceptibilities to Ca2+ overload Am J Physiol Heart Circ Physiol 2006 290 5 H2155 2162 10.1152/ajpheart.00958.2005 16399865

[B14] Ander BP Hurtado C Raposo CS Maddaford TG Deniset JF Hryshko LV Pierce GN Lukas A Differential sensitivities of the NCX1.1 and NCX1.3 isoforms of the Na+-Ca2+ exchanger to alpha-linolenic acid Cardiovasc Res 2007 73 2 395 403 10.1016/j.cardiores.2006.09.013 17059813

[B15] Web-based VISTA software http://genome.lbl.gov/vista/index.html

[B16] University of California Santa Cruz (UCSC) Genome Browser http://genome.ucsc.edu/

[B17] Janosikova B Pavlikova M Kocmanova D Vitova A Vesela K Krupkova L Kahleova R Krijt J Kraml P Hyanek J Genetic variants of homocysteine metabolizing enzymes and the risk of coronary artery disease Mol Genet Metab 2003 79 3 167 175 10.1016/S1096-7192(03)00079-9 12855221

[B18] Kepp K Juhanson P Kozich V Ots M Viigimaa M Laan M Resequencing PNMT in European hypertensive and normotensive individuals: no common susceptibilily variants for hypertension and purifying selection on intron 1 BMC medical genetics 2007 8 47 10.1186/1471-2350-8-47 17645789 PMC1947951

[B19] International Diabetes Federation http://www.idf.org/

[B20] United Laboratories, Tartu University Hospital http://www.kliinikum.ee/verekeskus/

[B21] Diagnostics Division Laboratory, the North Estonia Medical Centre http://www.regionaalhaigla.ee/?op=body&id=50/

[B22] Genepop web Version 3.4 http://genepop.curtin.edu.au/

[B23] PLINK software http://pngu.mgh.harvard.edu/~purcell/plink/

[B24] Wu J Province MA Coon H Hunt SC Eckfeldt JH Arnett DK Heiss G Lewis CE Ellison RC Rao DC An investigation of the effects of lipid-lowering medications: genome-wide linkage analysis of lipids in the HyperGEN study BMC Genet 2007 8 60 10.1186/1471-2156-8-60 17845730 PMC2045675

[B25] Tobin MD Sheehan NA Scurrah KJ Burton PR Adjusting for treatment effects in studies of quantitative traits: antihypertensive therapy and systolic blood pressure Stat Med 2005 24 19 2911 2935 10.1002/sim.2165 16152135

[B26] Web-based analysis tool ClustalW2 http://www.ebi.ac.uk/Tools/clustalw2/index.html

[B27] Haploview software http://www.broadinstitute.org/haploview/haploview/

[B28] Rowntree RK Vassaux G McDowell TL Howe S McGuigan A Phylactides M Huxley C Harris A An element in intron 1 of the CFTR gene augments intestinal expression in vivo Hum Mol Genet 2001 10 14 1455 1464 10.1093/hmg/10.14.1455 11448937

[B29] Seshasayee D Geiger JN Gaines P Wojchowski DM Intron 1 elements promote erythroid-specific GATA-1 gene expression J Biol Chem 2000 275 30 22969 22977 10.1074/jbc.M002931200 10811657

[B30] Warnecke C Willich T Holzmeister J Bottari SP Fleck E Regitz-Zagrosek V Efficient transcription of the human angiotensin II type 2 receptor gene requires intronic sequence elements Biochem J 1999 340 Pt 1 17 24 10.1042/0264-6021:3400017 10229654 PMC1220217

[B31] Eddy J Maizels N Conserved elements with potential to form polymorphic G-quadruplex structures in the first intron of human genes Nucleic Acids Res 2008 36 4 1321 1333 10.1093/nar/gkm1138 18187510 PMC2275096

[B32] Chen FC Chen CJ Li WH Chuang TJ Human-specific insertions and deletions inferred from mammalian genome sequences Genome Res 2007 17 1 16 22 10.1101/gr.5429606 17095709 PMC1716262

[B33] Tian D Wang Q Zhang P Araki H Yang S Kreitman M Nagylaki T Hudson R Bergelson J Chen JQ Single-nucleotide mutation rate increases close to insertions/deletions in eukaryotes Nature 2008 455 7209 105 108 10.1038/nature07175 18641631

[B34] Lercher MJ Hurst LD Human SNP variability and mutation rate are higher in regions of high recombination Trends Genet 2002 18 7 337 340 10.1016/S0168-9525(02)02669-0 12127766

[B35] Ball EV Stenson PD Abeysinghe SS Krawczak M Cooper DN Chuzhanova NA Microdeletions and microinsertions causing human genetic disease: common mechanisms of mutagenesis and the role of local DNA sequence complexity Hum Mutat 2005 26 3 205 213 10.1002/humu.20212 16086312

[B36] Chuzhanova NA Anassis EJ Ball EV Krawczak M Cooper DN Meta-analysis of indels causing human genetic disease: mechanisms of mutagenesis and the role of local DNA sequence complexity Hum Mutat 2003 21 1 28 44 10.1002/humu.10146 12497629

[B37] Kofuji P Lederer WJ Schulze DH Mutually exclusive and cassette exons underlie alternatively spliced isoforms of the Na/Ca exchanger J Biol Chem 1994 269 7 5145 5149 8106495

[B38] Liu R Paxton WA Choe S Ceradini D Martin SR Horuk R MacDonald ME Stuhlmann H Koup RA Landau NR Homozygous defect in HIV-1 coreceptor accounts for resistance of some multiply-exposed individuals to HIV-1 infection Cell 1996 86 3 367 377 10.1016/S0092-8674(00)80110-5 8756719

[B39] Samson M Libert F Doranz BJ Rucker J Liesnard C Farber CM Saragosti S Lapoumeroulie C Cognaux J Forceille C Resistance to HIV-1 infection in caucasian individuals bearing mutant alleles of the CCR-5 chemokine receptor gene Nature 1996 382 6593 722 725 10.1038/382722a0 8751444

[B40] Dhandapany PS Sadayappan S Xue Y Powell GT Rani DS Nallari P Rai TS Khullar M Soares P Bahl A A common MYBPC3 (cardiac myosin binding protein C) variant associated with cardiomyopathies in South Asia Nat Genet 2009 41 2 187 191 10.1038/ng.309 19151713 PMC2697598

[B41] Kasai T Miyauchi K Kubota N Tamura H Kojima T Yokoyama K Kurata T Daida H The relationship between the metabolic syndrome defined by various criteria and the extent of coronary artery disease Atherosclerosis 2008 197 2 944 950 10.1016/j.atherosclerosis.2007.08.023 18096168

[B42] Wassink AM Graaf Y van der Olijhoek JK Visseren FL Metabolic syndrome and the risk of new vascular events and all-cause mortality in patients with coronary artery disease, cerebrovascular disease, peripheral arterial disease or abdominal aortic aneurysm Eur Heart J 2008 29 2 213 223 10.1093/eurheartj/ehm582 18199567

[B43] Krawczak M Cooper DN Gene deletions causing human genetic disease: mechanisms of mutagenesis and the role of the local DNA sequence environment Hum Genet 1991 86 5 425 441 10.1007/BF00194629 2016084

