# Supplementary material for: Hypervariable intronic region in NCX1 is enriched in short insertion-deletion polymorphisms and showed association with cardiovascular traits
Source: BMC Med Genet. 2010 Jan 28;11:15. doi: 10.1186/1471-2350-11-15 (PMC2832636; doi:10.1186/1471-2350-11-15)
Supplement: Additional file 2 — Additional information for Materials and Methods. Additional information for Materials and Methods. [file 1471-2350-11-15-S2.PDF]

Hypervariable intronic region in *NCX1* is enriched in short insertion-deletion polymorphisms and showed association with cardiovascular traits

Katrin Kepp[1], Elin Org[1], Siim Sõber[1], Piret Kelgo[1], Margus Viigimaa[2], Gudrun Veldre[1,3], Neeme Tõnisson[1], Peeter Juhanson[1], Margus Putku[1], Andreas Kindmark[4], Viktor Kozich[5] and Maris Laan[1]*

Additional file 2

Additional information for Materials and Methods

DHPLC assay design and electrophoresis conditions

For the DHPLC primer design, the web-based Primer3 software was used [1]. All primer sequences were analyzed for potential multiple binding sites in the human genome using GenomeTester1.3 software [2]. The appropriate melting conditions for each of the selected DHPLC PCR fragments was calculated using web based DHPLC Melt Program [3]. DHPLC PCR primers and fragment characteristics are listed in additional file 1. PCR products were amplified by touchdown method using 50-100ng genomic DNA, Smart-Taq Hot DNA polymerase (Naxo OÜ, Tartu, Estonia), GeneAmp PCR System 2700 thermal cycler (Applied Biosystems Inc., USA); and the amplification conditions are previously described [4]. The DHPLC analyses and quality control for every assay were performed according to the manufacturer’s recommendations (DHPLC; Wave Technologies Inc. USA).

DGGE genotyping assay

The DGGE PCR primers (forward/reverse primers: 5'- GGATTGAGCCCTGTCACTGA -3'/ 5'- **(**CGCCCGCCGCGCCCCGCGCCCGGCCCGCCGCCCCCGCCCGGGGGAGAAAG ACACTGTTT- 3'; 327bp amplicon) were designed using the MELT Ingeny 1.0.1 software (Ingeny, Goes, Netherlands). Supplementing the 5' end of the reverse primer with a GC clamp (a sequence of 40 G and C deoxynucleotides) allowed the detection of single-base substitutions present in the fragment. PCR conditions were identical to the DHPLC analysis. DGGE electrophoresis by INGENY PhorU2 system was performed according to the manufacturer's instructions (Ingeny, Goes, Netherlands).

Subjects for association studies with cardiovascular disease

The HYPEST and CADCZ studies have been approved by the Ethics Committee on Human Research of University of Tartu, Estonia and by the Ethics Committee of Charles University–1st Faculty of Medicine, Prague, Czech Republic. All of the study participants are of Eastern European ancestry, have filled a self-administrated epidemiological questionnaire recording to their past and present health and life-style and have given their written informed consent. The HYPEST essential hypertension patients were selected based on the clinical diagnosis and profile of blood pressure specialists during the patients' ambulatory visits or hospitalization at the North Estonia Medical Center, Centre of Cardiology, or at the Cardiology Clinic, Tartu University Hospital, Estonia. The HYPEST healthy control cohort was recruited from among the long-term blood donors

across Estonia. All the included donors had no personal history of cardiovascular diseases and had also never been prescribed any relevant medications. In the current study, the essential hypertension patients (n=470) were defined as follows: (a) individuals diagnosed by blood pressure specialists; (b) subjects with prescribed antihypertensive medications; or (c) individuals with SBP>160 mmHg and DBP>100 mmHg readings. The matched normotensive control group (n=652) consisted of subjects with SBP<140 mmHg and DBP<90 mmHg.

The coronary artery disease (CAD) patients of the CADCZ study (n=296) were recruited by the Cardiology Department of the 2nd Clinic of Internal Medicine, Faculty Hospital Královské Vinohrady in Prague Czech Republic. 193 CAD patients had experienced myocardial infarction and 88 cases were additionally diagnosed with metabolic syndrome based on the criteria appointed by International Diabetes Federation [5]: triglycerides >1.7mmol/L; BMI>30kg/m^2; SBP>130mmHg; DBP>85mmHg; HDL >1.29mmol/L. The CADCZ controls were recruited in heath clinics across Czech Republic. They had no personal history of CAD, essential hypertension, MI, peripheral arterial disease, or stroke, and had never been prescribed any related medications. However, for 52 of 413 CAD controls included into the current study, the diagnosis of metabolic syndrome could not be excluded.

Reference for Additional file 2:

1. **Primer3 software** [http://frodo.wi.mit.edu/cgi-bin/primer3/primer3_www.cgi/]
2. **GenomeTester1.3 software** [http://bioinfo.ut.ee/genometester/]
3. **DHPLC Melt Program** [http://insertion.stanford.edu/melt.html]

4. Hallast P, Nagirnaja L, Margus T, Laan M: **Segmental duplications and gene conversion: Human luteinizing hormone/chorionic gonadotropin beta gene cluster**. *Genome Res* 2005, **15**(11):1535-1546.

5. **International Diabetes Federation** [http://www.idf.org/]
