# Supplementary figures and images for: Hypervariable intronic region in NCX1 is enriched in short insertion-deletion polymorphisms and showed association with cardiovascular traits
Source: BMC Med Genet. 2010 Jan 28;11:15. doi: 10.1186/1471-2350-11-15 (PMC2832636; doi:10.1186/1471-2350-11-15)

Additional Figure

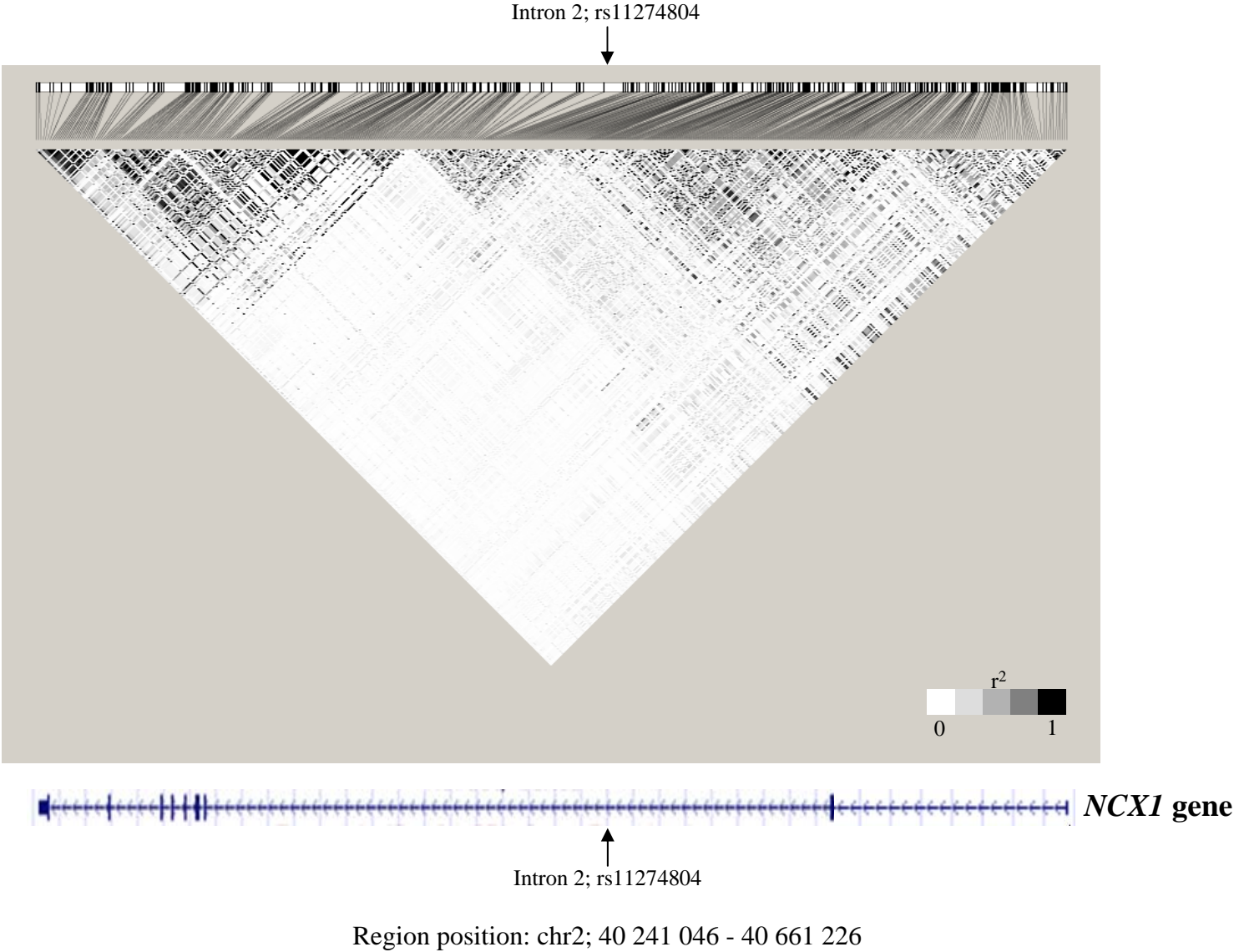

Supplement: Additional file 3 — LD structure of the human NCX1 gene 348 bp region. LD structure of the human NCX1 gene region (chr2; position: 40 241 046-40 661 226) shown as r2-blot. Upper white bar marks the positions of HapMap SNPs. Both arrows indicate the location of 14 bp indel (rs11274804) between two LD-blocks in the second intron of the NCX1 gene. [file 1471-2350-11-15-S3.PDF]
